# Multifaceted autoimmunity: new challenges and new approaches

**DOI:** 10.1016/j.ebiom.2023.104474

**Published:** 2023-02-09

**Authors:** eBioMedicine

The term autoimmunity refers to a multitude of syndromes and diseases that affect almost all human organs and tissues, from the skin to reproductive organs to the central nervous system. Autoimmune diseases can be systemic, as in systemic lupus erythematosus (SLE), rheumatoid arthritis, and scleroderma, or tissue-specific, as in Graves’ disease and type 1 diabetes. The apparent worldwide increase in the prevalence and incidence of autoimmune diseases (summarised in *Curr Opin Immunol*, Feb, 2023) emphasises the need for renewed efforts in a field with more questions than answers.

The autoimmunity conundrum has been further complicated by the emergence of SARS-CoV-2 in late 2019 and the resulting COVID-19 pandemic. The relationship between autoimmunity and COVID-19 is being investigated from numerous perspectives, all of which are difficult to cover in detail in a short editorial. Risk of severe COVID-19 is increased in patients with rheumatoid arthritis, especially in the subset with interstitial lung disease *(Lancet Rheumatology*, Nov, 2022). Treatments commonly used to treat autoimmune diseases could reduce the humoral responses to SARS-CoV-2, as discussed in the *Lancet Regional Health Western Pacific* (Dec, 2022), the *Lancet Gastroenterology and Hepatology* (Dec, 2022), and *eBioMedicine* (July, 2022). Autoimmunity might also affect the development of post-acute sequelae SARS-CoV-2 infection (usually referred to as long COVID); positive antinuclear antibodies were associated with persistent symptoms 12 months after COVID-19 in a small, preliminary report (*Eur Resp J*, Aug, 2022).

One of the hardest questions to assess is whether SARS-CoV-2 infections, even mild or asymptomatic ones, can trigger or alter the clinical course of autoimmune disease. A large retrospective study of electronic health records in our sister journal *eClinicalMedicine* (Feb, 2023) suggested increased risk of several autoimmune disease in people with positive PCR (n = 888,463) versus people with negative PCR (n = 2,926,016) for SARS-CoV-2. The reported hazard ratios were lower for inflammatory bowel disease, and higher for rheumatoid arthritis, SLE, and ankylosing spondylitis.

Although major clinical and basic research efforts are invested in these issues, we should consider that health outcomes in autoimmunity can be affected not only by biological factors, but also by environmental and societal influences. An observational study in the *Lancet Rheumatology* (Sept, 2022) collated data from 14 044 patients with diagnosed rheumatic disease from 23 countries collected from the COVID-19 Global Rheumatology Alliance registry between March 12, 2020, and Aug 27, 2021. The study highlighted the associations of air pollution, population mobility, and proportion of the population older than 65 years with increased odds of mortality. Conversely, number of hospital beds, human development index, stringent government responses, and longer follow-ups were associated with decreased odds of mortality. Thus, addressing regional disparities might require efforts targeting societal and environmental factors as well as the biology of the individual patients.

Precision medicine is leading to multiple advances in the field of autoimmunity. Clinical responses to rituximab, an agent commonly used in rheumatoid arthritis and other autoimmune diseases, vary widely. A longitudinal cohort study in *eBioMedicine* (Dec, 2022) showed that in 835 patients with rheumatoid arthritis or SLE, increased copies of the *FCGR3A*-158V allele (and therefore higher affinity for IgG1) are associated with increased odds of major clinical response at 6 months, with implications for personalised treatment. Substantial efforts are also made in characterising novel markers for improved stratification and prognosis. In type 1 diabetes, serum protein disulphide isomerase A1 levels might reflect pancreatic β cell stress and are increased in newly diagnosed (ie, within 48 h of blood collection), hospitalised children compared with healthy control individuals (*eBioMedicine*, Jan, 2023). In primary Sjogren's syndrome, T-cell receptor diversity was shown to be related to a subset of symptoms and autoantibody levels (*eBioMedicine*, Oct, 2022).

Because of the chronic nature of autoimmune diseases and the associated high burden of monitoring and treatment, there is a need for novel approaches to care. A randomised, double-blind, placebo-controlled, proof of concept trial (TREAT EARLIER, Leiden, Netherlands) evaluated whether early intervention in the preceding phases of arthralgia and subclinical joint inflammation could prevent the development of clinical rheumatoid arthritis or reduce the disease burden (*The Lancet*, July 23, 2022). 236 people with arthralgia clinically suspected of progressing to rheumatoid arthritis and MRI-detected subclinical joint inflammation were randomly assigned to a single intramuscular glucocorticoid injection (120 mg) and a 1-year course of oral methotrexate (up to 25 mg per week) or placebo, and were followed up for 1 year after treatment completion. Development of clinical arthritis, the primary endpoint, did not differ between the groups. However, MRI-detected inflammation, symptoms including pain and morning stiffness of the joints, and impairments (eg, presenteeism) showed sustained improvements in the intervention group. Although larger and longer studies are warranted, these findings suggest that altering the course of autoimmune diseases with early interventions might be possible.

We would also like to bring attention to less prevalent autoimmune diseases as Feb 28 is a day devoted to increasing awareness of rare diseases. By some counts there are more than 140 autoimmune diseases, and many of these fit the US National Institutes of Health definition of a rare disease (ie, affecting fewer than 1 in 2000 individuals). *The Lancet Rheumatology* (Apr, 2022) highlighted the multiple challenges patients with rare autoimmune disorders experience. These challenges include long so-called diagnostic odysseys, missing opportunities for interventions (especially important for children), the unfamiliarity of general practitioners, and little access to specialist care. On a positive note, strength-in-numbers approaches, as advocated by the UK Rare Autoimmune Rheumatic Disease Alliance, and use of novel technologies, as in the Platform Vector Gene Therapy (PaVe-GT) Pilot Project by the US National Center for Advancing Translational Sciences, could offer opportunities for innovation to finally start overcoming the issues of rarity.

The editorial team at *eBioMedicine*, part of Lancet Discovery Science, welcomes mechanistic, translational, and early clinical studies in autoimmunity and is invested in providing a forum for rare disease research.

